# Programme of Meeting of Medical Association of Georgia

**Published:** 1890-04

**Authors:** 


### PROGRAMME

### OF MEETING OF MEDICAL ASSOCIATION OF GEORGIA, AT BRUNSWICK, GA., APRIL 16TH, 17TH AND 18TH, 1890.

WEDNESDAY, APRIL 16TH, 1890. 1. Meeting called to order at 10:30 A. M., promptly, by retiring president, Dr. J. S. Todd, Atlanta, Ga. 2. Prayer by ——— 3. Address of welcome in behalf of Brunswick, by ——— 4. Response, by Dr. G. W. Mulligan, Washington, Ga. 5. Address, by President J. B. S. Holmes, Rome, Ga. 6. Report of committee on programme. 7. Announcing members of Board of Censors. 8. Applications for membership. 9. Report of Secretary. 10. Report of Treasurer. 11. Adjournment.

In the afternoon the Association will be entertained by the local committee in an excursion around the Harbor, spiced with a clam bake and oyster roast.

THURSDAY, APRIL 17TH, 1890. 1. Meeting to be called to order at 9 o'clock, A. M., promptly. 2. Reading of minutes. 3. Application for membership, 4. Report of Board of Censors. 5. At 10 o'clock, A. M., reading of paper, by Dr. S. C. Benedict, Athens, Ga., "Aseptic vs. Antiseptic Surgery." Leaders in discussion of this paper: Drs. Henry F. Campbell, W. F. Westmoreland, Robert Battey, Eugene Foster, W. F. Holt. 6. At 11 o'clock, A. M., paper by Dr. H. McHatton, Macon, Ga., "Railroad Surgery." Leaders in discussion of this paper: Drs. T. M. Holmes, W. P. Nicholson, A. P. Taylor, P. L. Hillsman, W. F. Westmoreland, Jr. 7. Twelve o'clock, M., Orator's address, by Dr. W. F. Westmoreland, Jr., Atlanta, Ga. 8. Adjournment, after orator's address. 9. Meeting to be called to order promptly at 3:30, P. M. 10. Appointment of Nominating Committee. 11. Applications for membership. 12. Four o'clock, P. M., reading of paper by Dr. K. P. Moore, Macon, Ga., "The Female Urethra, a source of trouble liable to be overlooked in our gynæcological investigations." Leaders of discussion: Drs. J. G. Earnest, W. A. Love, V. O. Hardon, G. H. Noble, R. J. Nunn. 13. Five o'clock, P. M., reading of paper by Dr. G. H. Noble, Atlanta, Ga., "Hyperæsthetic Endometritis." Leaders of discussion: Drs. J. W. Bailey, Robert Battey, M. J. Hatch, K. P. Moore. 14. Six o'clock, P. M., reading of paper by Dr. J. M. Hull, Augusta, Ga., "Rare Experience with Erysipelas in Eye Surgery." Leaders of discussion: Drs. S. B. Hawkins, W. S. Elkin, B. R. Doster, W. O'Daniel.

FRIDAY, APRIL 18th, 1890.—1. Meeting to be called to order, promptly at 9 o'clock, A. M. 2. Reading of minutes. 3. Report of Nominating Committee. 4. Report of Committee to audit Treasurer's books. 5. Report of Committee to audit Secretary's books. 6. Ten o'clock, A. M., reading paper by Dr. H. J. Williams, Macon, Ga., "The Importance of Chemical and Microscopical Examination of the Urine." Leaders of discussion: Drs. G. W. Mulligan, H. Burford, W. D. Bizzell, H. McHatton, R. J. Nunn. 7. At 11 o'clock, A. M., reading paper by Dr. R. O. Cotter, of Macon, Ga., "Progress of Ophthalmology and Rhinology." Leaders of discussion: Drs. J. M. Hull, A. W. Calhoun, T. M. McIntosh. 8. At 12 o'clock, M., reading of paper by Dr. A. C. Davidson, Sharon, Ga., "Cardiac Neurasthenia." Leaders of discussion: Drs. J. F. Lancaster, A. W. Griggs, J. S. Todd, J. A. Dunwody. 9. Adjourn at 1 o'clock, P. M. 10. Meeting called to order at 3:30 o'clock, P. M. 11. Reading paper by Dr. R. O. Engram, Montezuma, Ga., "Stricture of Male Urethra, and some form of Neurosis." Leaders of discussion: Drs. T. O. Powell, W. D. Bizzell, J. M. Gaston, S. C. Benedict. 12. At 4:30, P. M., reading of paper by Dr. J. A. Butts, "Climate of Brunswick, Ga." Leaders of discussion: Drs. A. C. Blain, P. R. Cortelyou, J. C. LeHardy. 13. Unfinished business. 14. New business. 15. Adjournment.

REGULATIONS.

No member can consume more than twenty minutes in reading a paper, and those discussing a paper will be limited to five minutes each.

While the Committee on Programme has appointed members to discuss papers, any member has the right to join in the discussions. Nor does this programme bar any member from presenting a voluntary paper.

If you are to read a paper, don't forget the following law governing the subject:

"No paper shall be read before this Association, by title or otherwise, until a complete copy of such paper shall have been placed in the hands of the Secretary for publication in the Transactions."

Please carefully examine the programme, and if your name is down to read a paper, or lead the discussion of one, be on hand at the time it is to be read.

In order to keep full meetings the Nominating Committee is requested to meet at night, second day.

The Association will meet daily at 9 A. M., adjourn at 1 P. M. o'clock. Assemble at 3:30 P. M., adjourn at 7 P. M.

EUGENE FOSTER, M. D.,
T. M. HOLMES, M. D., } Programme Committee
J. F. LANCASTER, M. D.

---

MACON, GA., March 10th, 1890.

DEAR DOCTOR: In extending to you the Annual Notice and invitation to attend the approaching meeting of the Medical Association of Georgia, at Brunswick, Ga., April 16-18, inclusive, it affords me pleasure to state, that the outlook for a large and interesting meeting is unusually promising. The Local Committee of Arrangements, at Brunswick, together with all her physicians and citizens, are actively at work, not only to give the members a cordial greeting and royal reception, but are also endeavoring to stir up the whole Profession of Southern Georgia; and we hope to have a large accession of new members. From the programme, which I have the pleasure to hand you, you will see that the Programme Committee has been active in securing papers for, and arranging an attractive and interesting programme. Other voluntary papers and discussions, than those on the programme, will be presented, and we feel safe in predicting a profitable and interesting meeting.

Reduced railroad rates have been secured on all the roads of the State on the certificate plan. Be sure to secure from the agent, where you purchase the going ticket, a certificate, which, after being signed by the Secretary of the Association, will entitle you to return on one-third the regular fare. If tickets are purchased at intermediate stations, buy only to the first terminal office, and then purchase ticket and get certificate from there to Brunswick.

Brunswick, Georgia's "City by the Sea," is the most rapidly growing city in Georgia, and will be a delightful place to visit at this season of the year. And I predict an exceedingly pleasant visit to all who may attend.

The physicians of Brunswick will tender the Association an excursion around the harbor, and treat the inland Doctors with a "Clam Bake" and an "Oyster Roast," besides giving a Grand Banquet on the second evening of the session.

Respectfully and fraternally,
K. P. MOORE,
Secretary.

